# Laparoscopic Rectopexy for Neorectal Prolapse after Robot-Assisted Intersphincteric Resection for Low Rectal Cancer: A Case Report

**DOI:** 10.70352/scrj.cr.26-0109

**Published:** 2026-05-09

**Authors:** Yuzo Harada, Shigenobu Emoto, Hiroaki Nozawa, Kazuhito Sasaki, Koji Murono, Yuichiro Yokoyama, Yuzo Nagai, Takahide Shinagawa, Yuichi Tachikawa, Satoshi Okada, Hiroshi Shiratori, Naoyuki Umetani, Soichiro Ishihara

**Affiliations:** 1 Department of Surgical Oncology, The University of Tokyo, Tokyo, Japan; 2 Department of Gastroenterological Surgery, Kawakita General Hospital, Tokyo, Japan

**Keywords:** laparoscopic rectopexy, neorectal prolapse, intersphincteric resection

## Abstract

**INTRODUCTION:**

Intersphincteric resection (ISR) is a sphincter-preserving procedure for lower rectal cancer; however, it can cause neorectal prolapse. Transanal repairs are commonly described but may fail, while transabdominal approaches are less frequently reported because of adhesions, bowel injury, and stoma risks. We present a patient with neorectal prolapse after robot-assisted ISR in whom transanal repair failed and subsequent laparoscopic rectopexy achieved a successful outcome, with a review of the literature.

**CASE PRESENTATION:**

A 72-year-old male patient underwent robot-assisted partial ISR with an ileostomy for lower rectal cancer. Neorectal prolapse and a posterior subcutaneous bulge appeared 1 month after surgery. Despite transanal repair using the Delorme procedure, 18 months after the initial surgery, the prolapse recurred within 1 month. Therefore, a laparoscopic rectopexy was performed. Adhesions around the neorectum were lysed, and the mobilization of the bowel was achieved until the anal canal. After confirming that cranial traction resolved both the neorectal prolapse and the bulge, a mesh was placed behind the bowel, and the seromuscular layer was fixed to the mesh and pelvis. The postoperative course was uneventful and no recurrence was observed during the 6-month follow-up.

**CONCLUSIONS:**

Although transanal repair is commonly performed after ISR due to concerns regarding adhesions and bowel injury, this case suggests that laparoscopic fixation can be effective in selected patients.

## Abbreviations

CCFIS
: Cleveland Clinic Florida Fecal Incontinence Score
ISR
: intersphincteric resection
TaTME
: transanal total mesorectal excision

## INTRODUCTION

ISR is a sphincter-preserving procedure for lower rectal cancer; however, neorectal prolapse is a recognized postoperative complication. Various studies have described transanal repair techniques, including the Delorme and Gant–Miwa–Thiersch procedures. However, these approaches are not always effective. In contrast, evidence regarding transabdominal repair remains limited owing to concerns regarding postoperative adhesions, bowel injury, and the risk of permanent stoma creation. We encountered a patient who developed neorectal prolapse after robot-assisted ISR. Considering these risks, we initially performed a transanal repair; however, this intervention was ineffective. Therefore, we performed laparoscopic rectopexy, resulting in a favorable outcome. Here, we report this case along with a review of the literature.

## CASE PRESENTATION

**Fig. 1:**
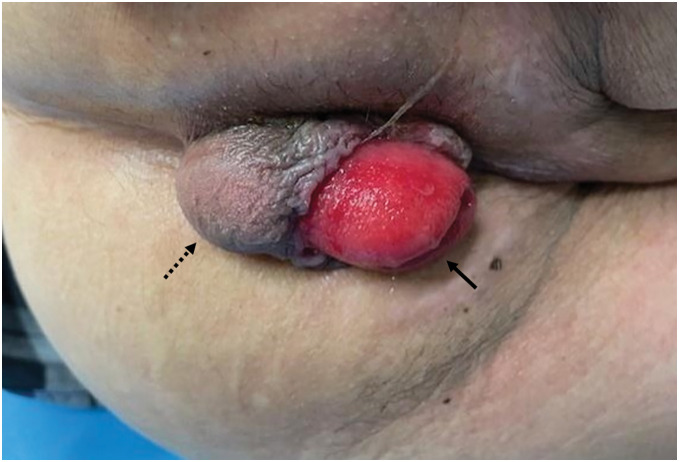
Neorectal full-thickness prolapse (arrow) and posterior subcutaneous bulge (dotted arrow).

A 72-year-old male patient underwent robot-assisted ISR with an ileostomy for lower rectal cancer. The patient’s BMI was 21.2 kg/m^2^, and his past medical history included diabetes mellitus, benign prostatic hyperplasia, prior right inguinal hernia repair, and prior surgery for a herniated intervertebral disc. The pathology revealed pT2, N1b, and pStage IIIa tumors (Union for International Cancer Control, 9th Edition). Neorectal prolapse and a posterior subcutaneous bulge appeared 1 month after the ISR (**Fig. 1**). Manual reduction was easy but was associated with pain. Stoma prolapse was also observed. After ileostomy closure, transanal repair using the Delorme procedure was performed 18 months after the ISR. The length of the prolapsed segment was approximately 3.5 cm. A circumferential mucosal incision was made proximal to the anastomosis, followed by submucosal dissection, plication of the muscle layer, resection of the mobilized mucosa, and mucosal suturing.

However, the prolapse recurred 1 month later, together with the reappearance of the bulge. Daily fecal incontinence required the use of pads, interfering with daily life (CCFIS 17). As the bulge was attributed to herniation of the proximal bowel beneath the posterior skin, transanal repair alone was unlikely to be effective; therefore, neorectal fixation was deemed essential. Consequently, we planned a laparoscopic rectopexy.

**Fig. 2:**
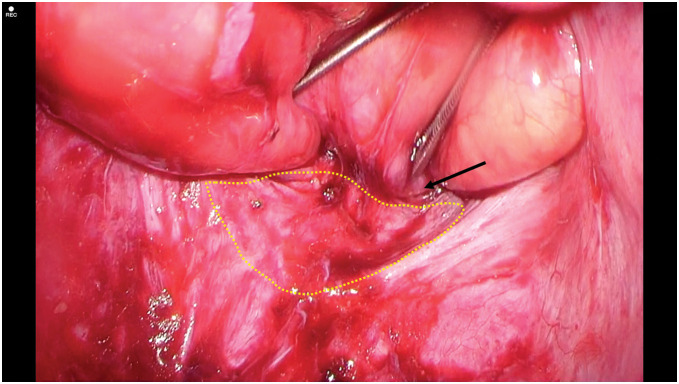
Intraoperative posterior view of the rectum following mobilization to the level near the anal canal. The arrow indicates the upper border of the anal canal, and the dotted outline denotes the site of proximal bowel herniation into the subcutaneous bulge.

**Fig. 3:**
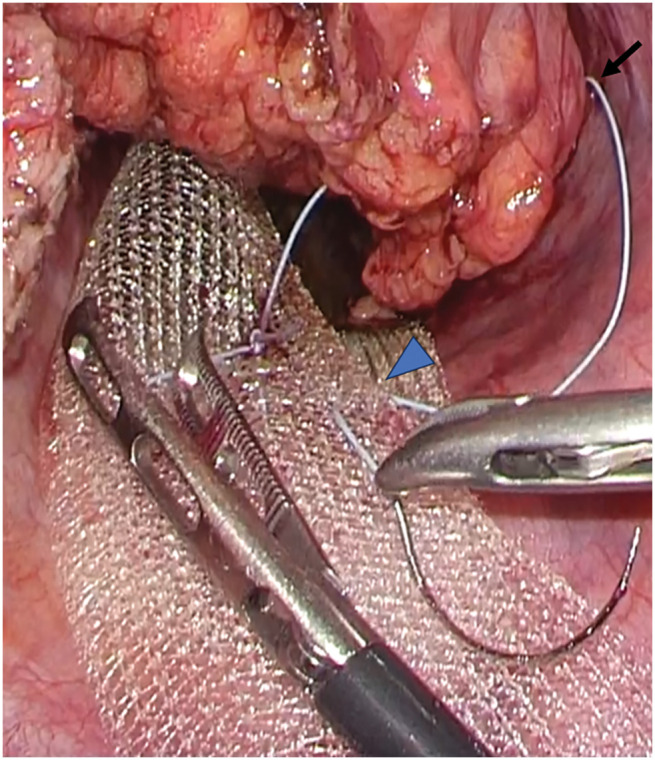
Meshes were placed anterior to the sacrum and fixed to the sacral periosteum at the promontory (arrowhead). The seromuscular layers of the right posterior rectal wall (arrow) and meshes were sutured together.

**Fig. 4:**
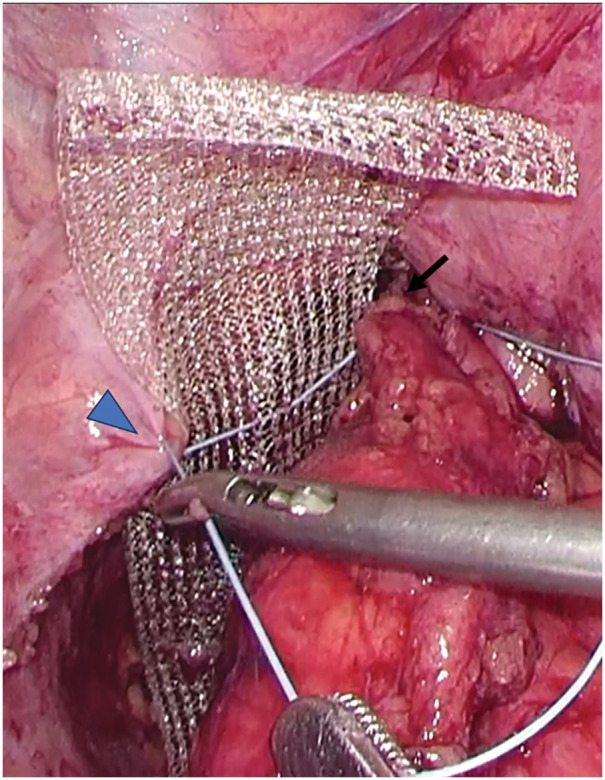
The seromuscular layers of the left rectal wall (arrow) and meshes (arrowhead) were sutured together.

**Fig. 5:**
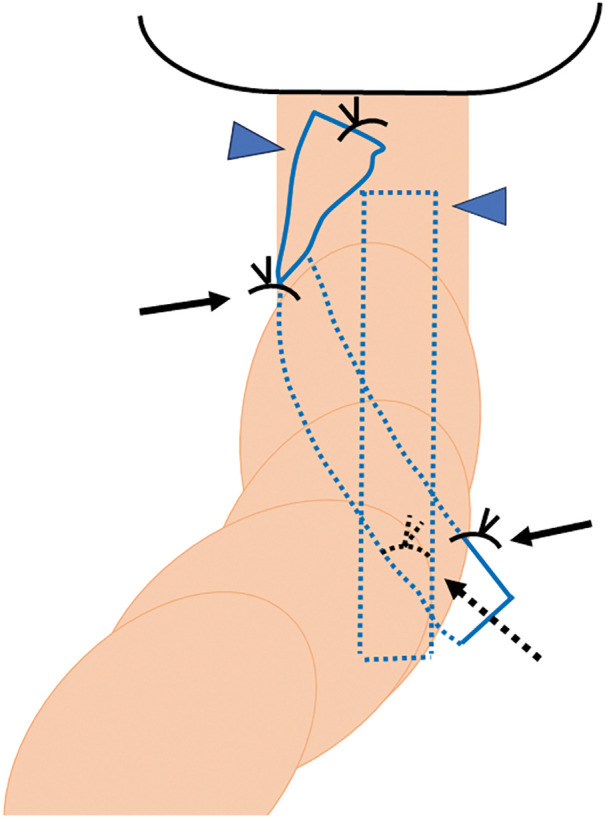
Neorectal prolapse repair with mesh rectopexy. Meshes 1 and 2 were placed anterior to the sacrum (arrowheads) and fixed to the sacral periosteum at the promontory (dotted arrow). The seromuscular layers of the bilateral rectal walls, mesh, and pelvic peritoneum were sutured together (arrow). The caudal end of mesh 1 was fixed to the anterior rectal wall.

The neorectum was redundant and descended deep into the pelvis. Due to prior surgery, it was adherent to the surrounding tissues. The adhesions were carefully lysed from the sacral promontory to the anal canal to avoid neorectal injury (**Fig. 2**). Digital rectal examination confirmed that cranial traction resolved both the prolapse and bulge. A trimmed 3 × 15 cm PROLENE mesh (Ethicon, Somerville, NJ, USA) (mesh 1) and another 3 × 12 cm mesh (mesh 2) were placed anterior to the sacrum and fixed to the sacral periosteum at the promontory (**Fig. 3**). The neorectum was elevated cranially, and the seromuscular layers of the bilateral rectal wall, mesh, and pelvic peritoneum were sutured (**Fig. 4**). The caudal end of mesh 1 was fixed to the anterior rectal wall (**Fig. 5**). The mesh was completely peritonealized using barbed sutures (3-0 V-Loc; Covidien, Mansfield, MA, USA). The operative time was 4 h and 59 min.

The postoperative course was uneventful, and the patient was discharged on POD 6. No recurrence was observed during the 6-month follow-up. Although the prolapse resolved, functional improvement was limited: the CCFIS improved from 17 to 15, and the maximum resting pressure increased slightly (22.5–28.5 mmHg).

## DISCUSSION

Rectal prolapse is associated with age-related pelvic floor weakening and increased abdominal pressure. Treatment options include transabdominal and transanal approaches. With the widespread use of laparoscopy, the number of laparoscopic abdominal procedures has increased, and favorable outcomes have been reported.^1)^

Neorectal prolapse is reported to occur in 15% of patients after ISR,^2)^ and resection of the internal sphincter, anal dilation for anastomosis, splenic flexure mobilization, history of pelvic surgery, and childbirth are reported to be risk factors.^3)^ Preventive considerations at the time of the initial ISR may reduce the risk of neorectal prolapse. Avoiding excessive redundancy of the proximal bowel, minimizing unnecessary mobilization such as extensive splenic flexure mobilization, and preserving pelvic supporting structures when oncologically feasible may be important.

Due to concerns about adhesions, bowel injury, and the risk of permanent stoma creation, transanal repair is frequently performed. Narihiro et al. repaired 33 patients using the Delorme procedure and reported a 15% recurrence rate; however, repeat Delorme repair was feasible.^4)^ Although transanal repair is considered safer after rectal surgery, it fails to improve fixation.

However, the transabdominal approach is considered more effective, it entails a higher surgical risk. Consequently, few reports have described transabdominal repair after rectal surgery. Nakamura et al. performed mesh repair for prolapse after partial ISR and argued that transanal repair can shorten the bowel but cannot improve fixation, making transabdominal repair more suitable in selected cases. They used mesh because of suspected tissue weakness related to age and diabetes.^5)^ Shapiro et al. and Landen also used mesh in a similar setting.^6,7)^ In contrast, Gys et al. avoided using mesh because future surgery for local recurrence might have been required.^8)^ Schulberg et al. treated recurrence after Altemeier repair following chemoradiotherapy and TaTME by robot-assisted fixation without a mesh, emphasizing preservation of the anastomosis and marginal vessels^9)^ (**Table 1**). Thus, optimal management remains unclear.

**Table 1:** Previously reported cases of transabdominal repair after rectal surgery

Authors	Year published	Age	Sex	BMI	Initial surgery	Initial surgery to prolapse	Prolapse repair	Mesh use	Recurrence	Follow-up period
Gys et al.^8)^	2018	68	Male	NR	TaTME	2 years	Posterior suturepexy	No	No	5 months
Shapiro et al.^6)^	2018	75	Female	NR	TaTME	NR	Ventral rectopexy	Yes	No	6 months
Schulberg et al.^9)^	2020	48	Male	NR	TaTME	4 months	Posterior suturepexy	No	No	6 months
Nakamura et al.^5)^	2022	70s	Female	18.1	Partial ISR	4 months	Posterior rectopexy	Yes	No	1 month
Landen^7)^	2023	46	Female	NR	Total ISR, J-pouch	1 year	Posterior rectopexy	Yes	No	1 year

ISR, intersphincteric resection; NR, not reported; TaTME, transanal total mesorectal excision

In our case, the transanal repair failed because the proximal bowel descended and adhered to the pelvis, causing a persistent posterior subcutaneous bulge. Although transabdominal surgery carries the risk of adhesiolysis-related bowel injury, we performed transabdominal repair. Adhesions from the previous robot-assisted ISR were not severe; however, adhesions around the neorectum required careful dissection. Although the sigmoid colon is usually shortened after rectal surgery, some patients retain a redundant sigmoid colon, depending on body habitus, which predisposes them to prolapse. In such patients, correcting the pelvic descent through mobilization and cranial suspension may be more effective, and a transabdominal approach may offer advantages. We used a mesh because we were concerned about tissue fragility given the history of recurrent prolapse and prior stoma prolapse, as described by Nakamura et al.^5)^. In the present case, complete peritonealization was achieved, which is important for minimizing mesh-related complications. Given the limited evidence, circumferential dissection to the anal canal after ISR likely resulted in near-complete loss of rectal support, leading to global pelvic descent of the neorectum. Although preservation of the lateral ligaments is considered an advantage of this technique in preventing postoperative constipation, this benefit was not applicable in this case because the lateral ligaments had already been transected during ISR. Nevertheless, we selected posterior mesh rectopexy because it provides firm fixation to the sacral promontory and allows effective cranial and posterior suspension of the neorectum. Ventral rectopexy might be considered unsuitable because of insufficient healthy anterior tissue. Another posterior rectopexy, such as the Wells modification, requires placement of a relatively wide mesh covering the posterior aspect of the rectum. In this case, we were concerned that a broad mesh could be difficult to completely peritonealize. Incomplete peritoneal coverage may increase the risk of adhesions and mesh-related complications.

Moreover, although the mesh was fixed to the sacral periosteum with a single stitch in this case, the mesh was securely anchored to the sacral periosteum with a deeply placed suture. Additionally, fixation to the neorectal seromuscular layer and pelvic peritoneum provided sufficient stabilization. This multipoint fixation may reduce the risk of mesh detachment or migration. Although the risk of mesh-related complications was acknowledged,^10)^ recurrence prevention was prioritized in this post-ISR setting, making this approach a reasonable option in carefully selected cases.

Although the prolapse was resolved, functional recovery was limited because of internal anal sphincter resection during ISR. In patients with marked postoperative dysfunction, surgery alone may be insufficient to improve the QOL. Adjunctive treatments, such as pelvic floor muscle training, biofeedback therapy, and sacral nerve stimulation, should be considered.

## CONCLUSIONS

Although transanal repair is commonly performed after ISR due to concerns regarding surgical risks, it may be insufficient when pelvic fixation is compromised. Laparoscopic rectopexy may be particularly effective in carefully selected patients, such as those in whom anterior support is insufficient and secure posterior fixation is required.

## DECLARATIONS

### Availability of data and materials

The data that support the findings of this study are available from the corresponding author upon reasonable request.
